# Resistance to the Cyclotide Cycloviolacin O2 in Salmonella enterica Caused by Different Mutations That Often Confer Cross-Resistance or Collateral Sensitivity to Other Antimicrobial Peptides

**DOI:** 10.1128/AAC.00684-17

**Published:** 2017-07-25

**Authors:** Sohaib Z. Malik, Marius Linkevicius, Ulf Göransson, Dan I. Andersson

**Affiliations:** a Department of Medical Biochemistry and Microbiology, Uppsala University, Biomedical Center, Uppsala, Sweden; b Division of Pharmacognosy, Department of Medicinal Chemistry, Uppsala University, Biomedical Center, Uppsala, Sweden

**Keywords:** antimicrobial peptide resistance, cyclotide, cycloviolacin O2, cross-resistance, collateral sensitivity, Salmonella enterica, antimicrobial peptides, fitness, mechanisms of resistance, Salmonella enterica serovar Typhimurium

## Abstract

Antimicrobial peptides (AMPs) are essential components of innate immunity in all living organisms, and these potent broad-spectrum antimicrobials have inspired several antibacterial development programs in the past 2 decades. In this study, the development of resistance to the Gram-negative bacterium-specific peptide cycloviolacin O2 (cyO2), a member of the cyclotide family of plant miniproteins, was characterized in Salmonella enterica serovar Typhimurium LT2. Mutants isolated from serial passaging experiments in increasing concentrations of cyO2 were characterized by whole-genome sequencing. The identified mutations were genetically reconstituted in a wild-type background. The additive effect of mutations was studied in double mutants. Fitness costs, levels of resistance, and cross-resistance to another cyclotide, other peptide and nonpeptide antibiotics, and AMPs were determined. A variety of resistance mutations were identified. Some of these reduced fitness and others had no effect on fitness *in vitro*, in the absence of cyO2. In mouse competition experiments, four of the cyO2-resistant mutants showed a significant fitness advantage, whereas the effects of the mutations in the others appeared to be neutral. The level of resistance was increased by combining several individual resistance mutations. Several cases of cross-resistance and collateral sensitivity between cyclotides, other AMPs, and antibiotics were identified. These results show that resistance to cyclotides can evolve via several different types of mutations with only minor fitness costs and that these mutations often affect resistance to other AMPs.

## INTRODUCTION

Antimicrobial peptides (AMPs) are highly potent, broad-spectrum, bactericidal compounds with membranes as targets, a feature that has led to a widespread belief that bacteria would have difficulties developing resistance to them (1); however, this belief has been challenged by a number of studies which demonstrate that AMP resistance is relatively easy to acquire (2–6). On the basis of the findings of these studies, it has been suggested that therapeutic exposure of bacteria to AMPs could result in the development of resistance, and as a consequence, we might compromise our innate immunity by equipping bacteria with the means to circumvent the defensive barrier posed by our own AMPs (7).

**FIG 1:**
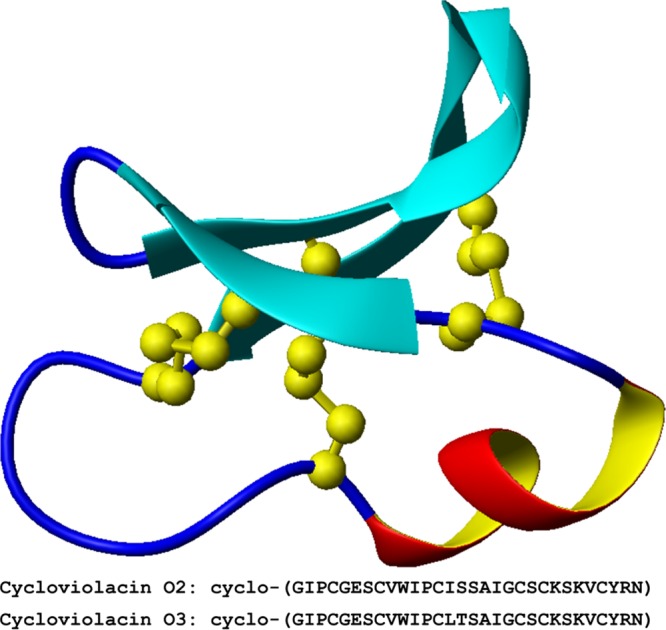
(Top) Schematic structure of the cyclotide scaffold. The disulfide bonds are shown in yellow (PDB accession number 2KNM). The α-helix in loop 3 is colored in red and yellow. (Bottom) The primary structures of the two cyclotides cyO2 and cyO3 are given.

Cyclotides constitute a family of plant miniproteins (8) with a cyclotide scaffold (Fig. 1), composed of a global structure with an interwoven network of three disulfide bonds (9). The resulting cyclic cystine knot is a very stable structure that can withstand harsh conditions (10). Cyclotides have various biological activities (11–16), but one less explored activity of cyclotides is their antibacterial action. Our previous work showed that the cyclotide cycloviolacin O2 (cyO2) is a potent AMP with activity against Gram-negative bacteria (17). Combined with their high stability and versatile backbone structure, this offered an argument to explore their antimicrobial potential. However, our current understanding of the bacterial mechanisms of resistance toward cyclotides is rudimentary, and any potential future use of these AMPs as drugs would require a better understanding of both mechanisms of action and resistance. Here, bacterial development of resistance to cyO2, isolated from Viola odorata, was studied.

## RESULTS

### Selection, isolation, and characterization of resistant mutants.

Salmonella enterica serovar Typhimurium (strain DA6192) was serially passaged in increasing concentration of cyO2. At the end of the experiment (100 to 150 passages), the 14 Salmonella lineages grew in the presence of 5 to 19 times the starting concentration of cyO2 (Table 1), indicating substantial increases in resistance. Clones used for further analysis were isolated from these populations by streaking for single colonies on LB agar (LA) plates.

**TABLE 1:** Summary of serial passaging experiment

*S*. Typhimurium lineage	No. of cycles	No. of generations (approximate)	Final concn of cyO2^*a*^ (μM)
1	133	798–931	70
2	146	876–1022	70
3	142	852–994	70
4	104	624–728	45
5	100	600–700	112.5
6	100	600–700	37.5
7	100	600–700	57.5
8	100	600–700	37.5
9	100	600–700	71
10	100	600–700	112.5
11	100	600–700	45
12	100	600–700	71
13	100	600–700	30.5
14	100	600–700	37.5

a The starting concentration of cyO2 was 6 μM.

Resistance in the isolated clones was characterized by time-kill experiments (data not shown) and MIC assays (Table 2). The *S*. Typhimurium mutants were 3- to 6-fold less susceptible to cyO2 than the wild type, and they suffered a fitness reduction of 19 to 35%, measured as the exponential growth rate in a rich medium and a minimal medium (Table 2).

**TABLE 2:** Relative fitness (in MHB and M9-G) and MIC of isolated mutants whose genomes were sequenced by whole-genome sequencing

*S*. Typhimurium strain	Isolated after cycle:	Relative fitness		cyO2 MIC (μM)
		MHB	M9-G	
DA6192 (wild type)	NA^*a*^	1	1	8.1
DA34444	45	0.80^*b*^	0.86^*b*^	37.5
DA34445	46	0.62^*b*^	0.75^*b*^	25
DA34446	80	0.82^*b*^	0.75^*b*^	50
DA34447	82	0.65^*b*^	0.72^*b*^	37.5
DA34448	126	0.81^*b*^	0.71^*b*^	37.5

a NA, not applicable.

b *P* < 0.05.

The whole genomes of five independent clones of *S*. Typhimurium resistant to cyO2 were sequenced and analyzed for sequence changes compared to the sequence of the wild type. All mutants had between 6 and 12 mutations in different genes (see Table SI2 in the supplemental material). Genes that were mutated in more than one mutant are listed in Table SI3.

### Reconstituted Salmonella mutants are resistant to cyO2.

Each of 10 candidate resistance mutations identified during the whole-genome sequencing were genetically reconstituted in a wild-type *S*. Typhimurium background by bacteriophage lambda Red recombineering (18). The criterion for the mutations to be selected as candidate mutations was their independent recovery in more than one serially passaged lineage, indicating that they were selected and conferred resistance rather than being randomly appearing mutations (Table SI3). The genes chosen for further study were *phoQ* and *pmrA*, which are involved in lipopolysaccharide biosynthesis (19); *envZ*, which encodes an inner membrane protein involved in regulation of expression of outer membrane proteins and virulence factors (20, 21); *ftsW*, which encodes a cell division protein and lipid flippase (22, 23); *yjeP*, which encodes a mechanosensitive ion channel (24); *rnc*, which encodes RNase III, which processes rRNAs and some mRNAs (25); *pnp*, which encodes an exoribonuclease which is a member of the mRNA degradosome (26); *rpoC*, which encodes the B′ subunit of RNA polymerase (27); and *plsX*, which has a role in phospholipid biosynthesis (28).

**FIG 2:**
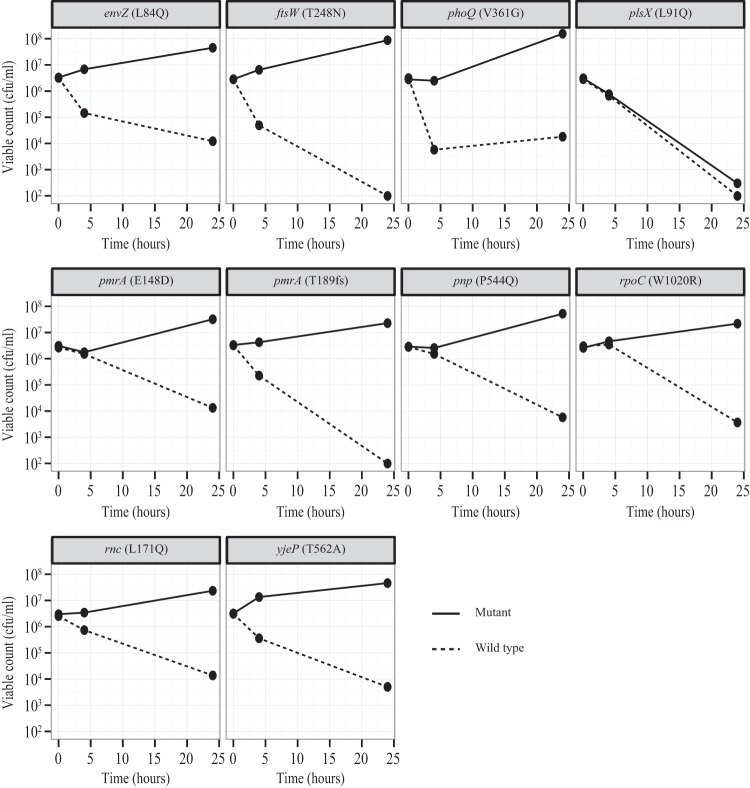
Killing curves of cyO2-resistant reconstituted mutants and their congenic wild-type strains after treatment with 12.5 μM cyO2. Bacteria were subjected to the peptide, and the number of viable cells was measured at 0, 4, and 24 h.

Reconstructed strains were tested for resistance to cyO2. Other than the *plsX* mutant, all reconstituted strains were less susceptible (3- to 5-log reduction in killing) to cyO2 than the congenic wild-type strains (Fig. 2).

### Combinations of resistance mutations confer a higher level of resistance.

**FIG 3:**
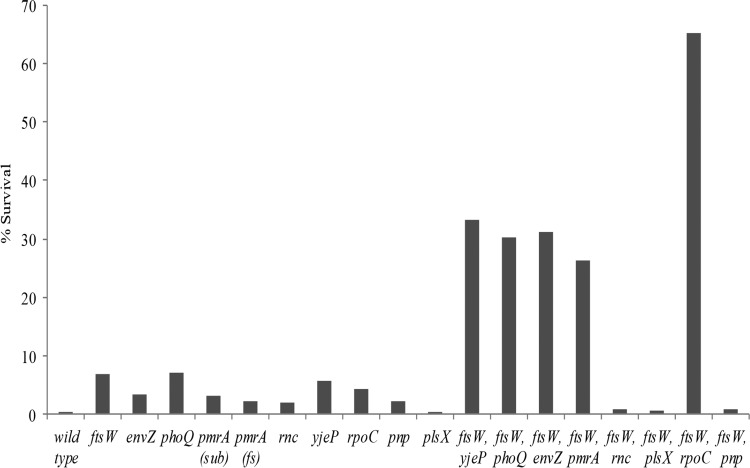
Survival index of the wild type and the cyO2-resistant single and double mutants. Bacteria were treated with 25 μM cyO2, and the number of surviving CFU was counted after 4 h. The number of surviving cells per milliliter was divided by the number of cells at the start of the experiment. The resulting value was expressed as a percentage.

Nine reconstituted mutants of *S*. Typhimurium showed reduced susceptibility to cyO2, but the resistance conferred by any single mutation alone could not explain the resistance levels observed in the original isolated mutants. Furthermore, as all cyO2-resistant mutants isolated from the serial passaging experiment contained more than six mutated genes, we combined the different resistance mutations to make double mutants. Since the *ftsW* mutation was repeatedly recovered in many lineages, all of the other resistance mutations were each combined with this mutation. These mutants were characterized by time-kill experiments and MIC assays to determine resistance levels (Fig. 3 and Table 3). The combination of the *ftsW* mutation with any other mutation (*envZ*, *phoQ*, *pmrA*, *yjeP*, *rpoC*, and *pnp*) resulted in increased resistance compared to that of the isolate with the single mutations, whereas the combination of *ftsW* with either *rnc* or *plsX* conferred no increased resistance.

**TABLE 3:** MIC values of cyO2-resistant double mutants

Strain	Gene mutation(s)	cyO2 MIC (μM)
DA6192	Wild type	8.1
DA34459	*ftsW*	10.1
DA39033	*ftsW*, *envZ*	18.7
DA39029	*ftsW*, *phoQ*	18.7
DA38894	*ftsW*, *pmrA*	15.6
DA38457	*ftsW*, *yjeP*	18.7
DA38459	*ftsW*, *rnc*	9.4
DA39980	*ftsW*, *rpoC*	37.5
DA39986	*ftsW*, *pnp*	25
DA38461	*ftsW*, *plsX*	9.4

### Fitness of cyO2-resistant mutants in laboratory media.

The fitness of the reconstituted cyO2-resistant mutants was measured in rich medium (Mueller-Hinton broth [MHB]) and minimal medium (M9 minimal medium plus 0.2% glucose [M9-G]) (Table 4). These mutants were grouped into three categories on the basis of fitness: (i) the two *pmrA* mutants and the *envZ* mutant showed no significant reduction in fitness in either of the two media, (ii) the *ftsW* mutant had a significant advantage over the wild type in both media, and (iii) the remaining six strains showed a significant fitness cost in both M9-G and MHB.

**TABLE 4:** Fitness of reconstituted strains relative to that of congenic wild-type strains

Strain	Mutation	Relative fitness^*a*^	
		MHB	M9-G
DA34459	*ftsW* (T248N)	1.01^*b*^	1.02^*b*^
DA38416	*envZ* (L84Q)	1	0.98
DA34453	*phoQ* (V361G)	0.96^*b*^	0.96^*b*^
DA38418	*pmrA* (T189fs)	0.99	1.01
DA34451	*pmrA* (E148D)	1.01	1
DA34455	*yjeP* (T562A)	0.96^*b*^	0.9^*b*^
DA34457	*rnc* (L171Q)	0.96^*b*^	0.91^*b*^
DA39973	*rpoC* (W1020R)	0.91^*b*^	0.93^*b*^
DA39977	*pnp* (P544Q)	0.97^*b*^	0.99^*b*^
DA34461	*plsX* (L91Q)	0.96^*b*^	0.98^*b*^

a Fitness relative to that of the congenic wild-type strains, which was set equal to 1.00.

b *P* < 0.05.

### Some cyO2-resistant mutants show a fitness advantage in mice.

**FIG 4:**
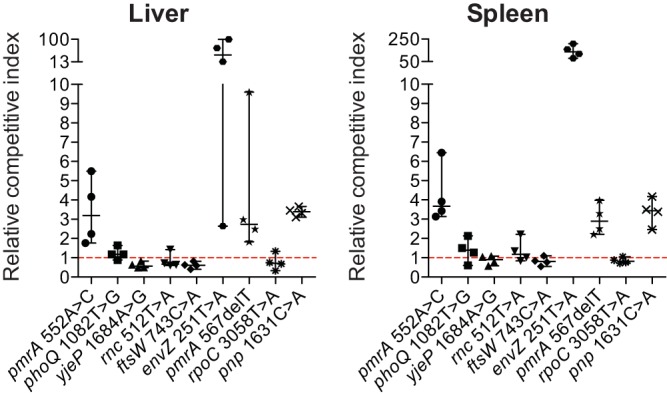
*In vivo* fitness of cyO2-resistant strains. cyO2-resistant strains were competed with their respective wild-type strains in a mouse infection model. The horizontal line for each time point represents the median value. Error bars indicate the range of data points. The relative competitive index values were normalized for the effect of ChlR and KanR genetic markers.

The fitness of reconstituted cyO2-resistant mutants was measured by a competition experiment in a mouse intraperitoneal infection model. As shown in Fig. 4, no fitness cost was observed for any of the mutants. In contrast, the *envZ* mutant, the *pnp* mutant, and two *pmrA* mutants had a significant fitness advantage over the wild type. In particular, the *envZ* mutant possessed an exceptionally high advantage over its congenic wild-type strain, where the ratio of mutant/wild type changed from 1:1 to from 100:1 to 200:1 in spleen and liver over only 2 days of growth in mice.

### Cross-resistance of cyO2-resistant mutants to cyclotides, AMPs, and antibiotics.

Cross-resistance to cyO3 was determined by time-kill experiments using the cyO2-resistant single mutants. Apart from one of the *pmrA* mutants, cyO2-resistant mutants were less susceptible to cyO3 than the wild type (Fig. SI1). However, the degree of reduced susceptibility at the tested concentration (12.5 μM) varied among the different mutants, and the most pronounced cross-resistance was seen in the *ftsW*, *phoQ*, and *rnc* mutants.

The cross-resistance of cyO2-resistant mutants toward other classes of AMPs was determined by MIC assays in microtiter plates, using three peptides: LL-37, which is a human cathelicidin; CNY100HL (CNY), which is a derivative of the C3a component of the human complement system; and wheat germ histones (WGH) (Table SI4). No significant increase in the MIC was observed for LL-37. Only the *pnp* mutant was less susceptible to CNY than the wild type, and a 2-fold increase in MIC values was seen for WGH in all mutants.

Cross-resistance to a set of antibiotics was determined using Etests. The *envZ* mutant was less susceptible to several antibiotics (Table SI5). In contrast, the *ftsW* mutant was slightly more susceptible to penicillin G and ampicillin than the wild type. Other small differences (up to 2-fold) in antibiotic susceptibility were observed for several different mutants (Table SI5).

### Cross-resistance of AMP-resistant mutants to cyO2.

**FIG 5:**
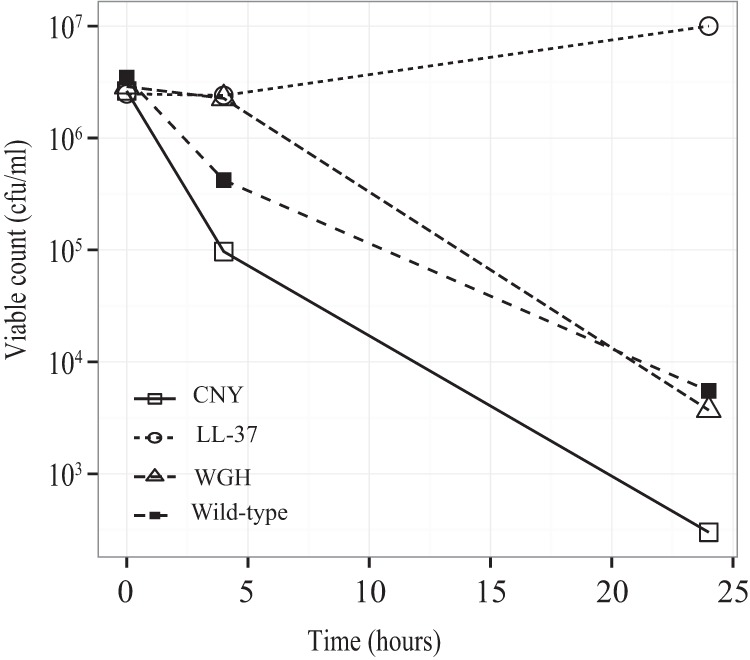
Susceptibility of *S*. Typhimurium mutants serially passaged with the AMPs or antibiotics indicated in the key to cyO2 at 12.5 μM.

**FIG 6:**
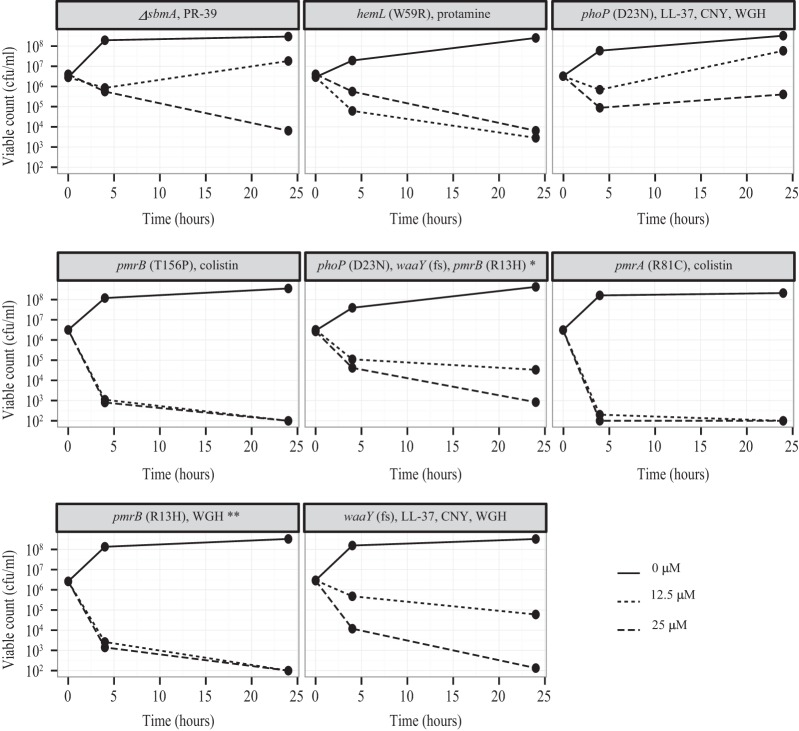
Cross-resistance profile of cyO2. In the title to each panel, the genotype and the peptide to which the tested strain is resistant are indicated. Samples were withdrawn at 0, 4, and 24 h. *, triple mutant (the triple mutant had resistance characteristics similar to those of the single mutants; **, very-low-level resistance.

A set of *S*. Typhimurium mutants isolated from different serial passaging experiments previously performed in the Department of Medical Biochemistry and Microbiology and with altered cell membrane characteristics were tested for cross-resistance to cyO2. As shown in Fig. 5, an LL-37-resistant mutant was cross-resistant to cyO2 (3 logs less killing than that of the wild type after 24 h). As these mutations were not in identical genetic backgrounds, these effects were explored further using a set of defined AMP-resistant strains constructed in other studies (9, 29–31). As can be seen in Fig. 6, an *sbmA* deletion mutant resistant to PR-39 and a *phoP* mutant resistant to LL-37, CNY, and WHG were cross-resistant to cyO2 (3 to 4 logs less killing after 24 h).

### AMP-resistant mutants that show collateral sensitivity to cyO2.

**FIG 7:**
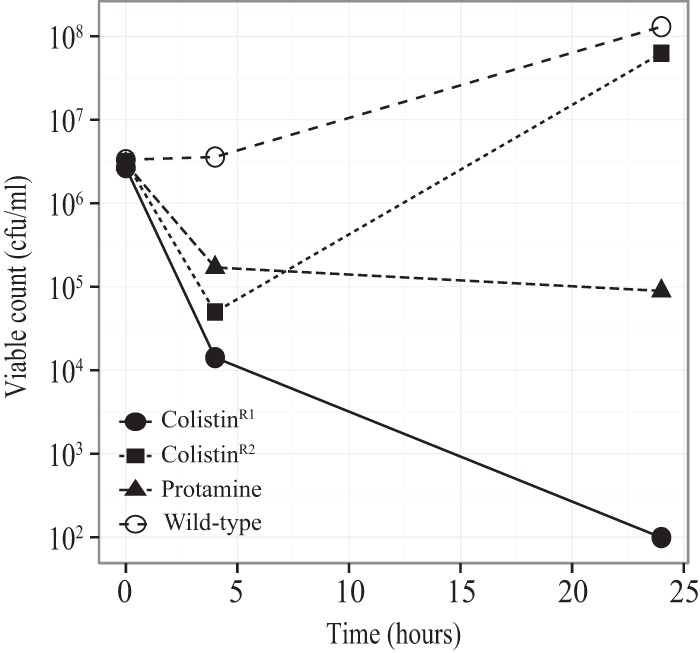
Collateral sensitivity to cyO2. Mutant strains resistant to other AMPs or antibiotics were treated with cyO2 at 6.25 μM. Samples were withdrawn at 0, 4, and 24 h.

As shown in Fig. 7, two cases of collateral sensitivity to cyO2 were identified. First, certain mutations in the *pmrAB* system conferring resistance to colistin rendered these mutants collaterally sensitive to cyO2. For example, one colistin-resistant *pmrA* mutant (R81C mutation, strain DA10857) was particularly susceptible to cyO2, showing 3 logs of killing after 4 h and complete killing after 24 h, whereas there was no killing for the wild type. However, it is notable that certain *pmrA* mutations did in fact confer resistance to cyO2 (Fig. 2), indicating that the *pmrA* mutations have allele-specific effects. Second, a protamine-resistant *hemL* mutant (W59R, DA11799) was collaterally more sensitive to cyO2 (3 logs more killing after 24 h).

## DISCUSSION

**FIG 8:**
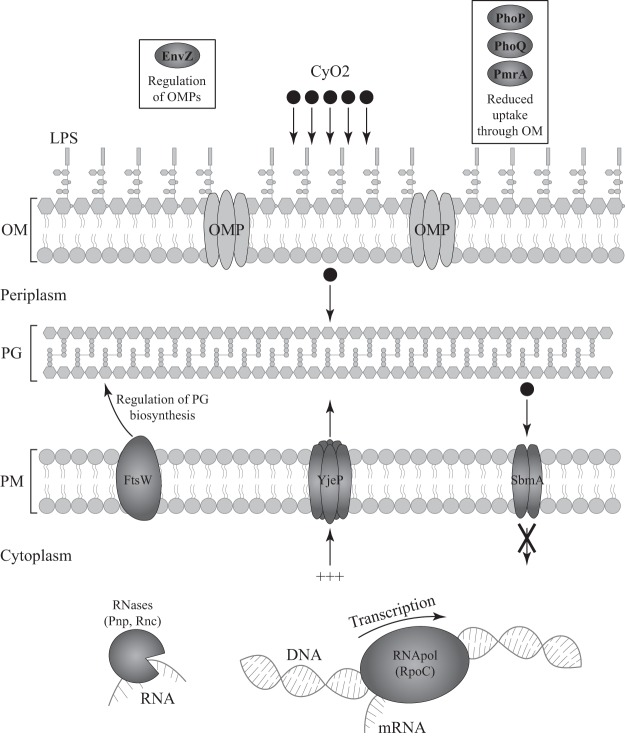
Potential pathways of resistance to cyO2. OMPs, outer membrane proteins; OM, outer membrane; LPS, lipopolysaccharide; PG, peptidoglycan; PM, plasma membrane.

The mutational pathways conferring resistance to cyO2 could be broadly divided into those regulated by (i) genes (*pmrA*, *phoPQ*, *envZ*, *rnc*, and *pnp*) that have previously been associated with the development of resistance to several AMPs, (ii) genes (*rpoC* and *sbmA*) that are involved in conferring resistance to AMPs that are internalized into the cytoplasm, and (iii) genes that have not been previously linked with the development of resistance to AMPs (*ftsW* and *yjeP*). The mutations that confer resistance to cyO2 are expected to change bacterial physiology in different ways (Fig. 8). It is notable that a number of the identified resistance mutations are in genes whose products regulate the expression of several other genes that not only are involved in antimicrobial peptide resistance but also confer protection against the innate host defense at large. Thus, these mutations could have broad effects and result in modification of lipopolysaccharide, secretion systems, biofilm formation, and the outer membrane protein profile. Such regulatory genes conferring resistance to cyO2 include (i) the transcriptional regulators *envZ*, *phoPQ*, and *pmrA*, which act either directly or through downstream response regulators (19, 21, 32), and (ii) the posttranscriptional regulators ribonucleases *rnc* and *pnp*, which control RNA turnover and the maturation of different virulence factors (Fig. 8) (33–35). Resistance mutations other than mutations in genes controlling these broad regulatory mechanisms are discussed below.

The mutation T248N in the essential cell division protein FtsW confers resistance to cyO2. It was present in four out of the five cyO2-resistant strains isolated. FtsW is proposed to be a lipid II flippase in Escherichia coli (23), and as a late recruit to the divisome, it has an important role in septum formation (36). This mutation might be involved in blocking the access of cyclotides to the phosphatidylethanolamine (PE) phospholipids, which are preferentially targeted by cyclotides (37, 38). Interestingly, the segment from E240 to A249 of the FtsW protein, which contains the cyO2 resistance mutation, is a key element in the functioning of FtsW in the septal peptidoglycan assembly machinery (39), which is assembled at about the time when PE phospholipids concentrate at the septum and required for constriction of the *ftsZ* ring (40).

Resistance to AMPs conferred by mutations in the putative membrane transporter of peptides SbmA (41) has been previously seen for AMPs that are internalized into the bacterial cytoplasm. This includes the proline-rich peptides, e.g., PR-39; the glycopeptide bleomycin; and the lassopeptides microcins J25 and B17 (29, 42, 43). This is in line with a previous observation that cyclotides are taken up into human lymphoma cells (44), implying that cyclotides have modes of action other than membrane damage.

A substitution mutation in *rpoC* (W1020R), which encodes the RNA polymerase B′ subunit, confers resistance to cyO2. Three of the five isolated *S*. Typhimurium mutants contained a mutation in *rpoC*, and two of these had the above-mentioned mutation (the other mutation was F1165S). Similar mutations in the central channel of RNA polymerase have been demonstrated to confer resistance to microcin J25 (45, 46).

Finally, a single nucleotide variation in the mechanosensitive ion channel *yjeP*, whose activity has been characterized in E. coli (24), conferred resistance to cyO2. This nonspecific ion channel responds to hypo-osmotic shock by releasing osmotically active ions and solutes, which might be a mechanism for compensation for the osmotic stress induced by cyO2.

Four of the nine reconstituted mutants carried a significant fitness advantage over their congenic wild-type strains in a mouse infection model, while the other five mutants were as fit as the wild type (Fig. 4). Generally, it is believed that AMP resistance is accompanied by an associated fitness cost. The significant fitness advantage acquired by almost half of the cyO2-resistant mutants in mice challenges this notion and suggests that AMP resistance might be associated with higher virulence in a host, a potential concern that has been pointed out previously (47).

In the double mutants, where one of the other resistance mutations was combined with the *ftsW* mutation, the general trend was that two mutations increased the level of resistance. One double mutant (the *ftsW rpoC* mutant) had a 4-fold increase in MIC compared to the wild type (Table 3). As demonstrated by the high number of different mutations that could be added to the *ftsW* mutation to increase the level of resistance, the potential mutational target for cyO2 resistance is large.

**FIG 9:**
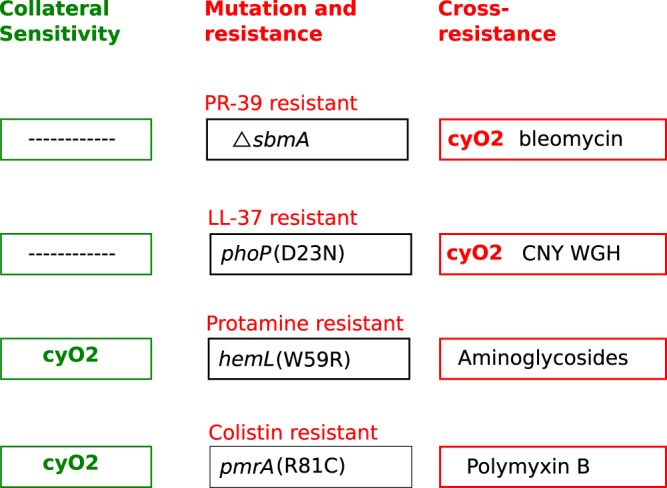
Resistance relationships between peptide antimicrobial agents.

As summarized in Fig. 9, collateral sensitivity and cross-resistance were common among the tested mutants. Thus, two cases of collateral sensitivity to cyO2 were observed: *pmrA* mutations selected for colistin resistance (30), and a *hemL* mutation selected for resistance to protamine (31). Conversely, several cases of cross-resistance were seen, where mutations in *sbmA*, *phoP*, *hemL*, and *pmrA* conferred resistance to AMPs and/or antibiotics other than those they were selected on.

In conclusion, the presented data demonstrate that several different types of mutations can generate resistance to cyclotides. These mutations are either neutral or confer a fitness advantage in a mouse infection model and often affect resistance to other AMPs by conferring either cross-resistance or collateral sensitivity. The finding of enhanced fitness in mice and cross-resistance suggests that the future therapeutic use of AMPs needs to be carefully monitored and evaluated to avoid the risk of selection of mutants with potential cross-resistance to human AMPs and/or antibiotics. However, the occurrence of collateral sensitivity is promising, since it suggests that an alternating drug-cycling protocol may slow the rate of development of resistance to AMPs, as has been observed for antibiotics (48, 49).

## MATERIALS AND METHODS

### Plant material.

Dried aerial parts of Viola odorata L. (Violaceae) were obtained from Alfred Galke (Gittelde, Germany; lot 17057).

### Extraction of cyclotides.

cyO2 and cyO3 (Fig. 1) were isolated as previously described (50).

### HPLC.

For fractionation of plant extracts, a Waters 600 controller high-performance liquid chromatography (HPLC) system was used (Waters Corporation, MA, USA). A Phenomenex C_18_ column (250 by 21.20 mm [inside diameter {i.d.}]; particle size, 10 μm; 300 Å) was used. A linear gradient from 10% acetonitrile (ACN), 0.05% trifluoroacetic acid (TFA) (buffer A) to 60% ACN, 0.05% TFA (buffer B) was run over 45 min. Fractions were analyzed by electrospray ionization-mass spectrometry (ESI-MS) in the positive-ion mode; the capillary temperature was set at 220°C, and the spray voltage was set at 4 kV. Purification of fractions was done by using an Äkta basic HPLC system (Amersham Biosciences, Uppsala, Sweden). A Phenomenex C_18_ column (250 by 10 mm [i.d.]; particle size, 5 μm; 300 Å) was used for purification. For purification of cyO2, reverse-phase HPLC was done by running a linear gradient from 40 to 70% buffer B in buffer A. Fractions were analyzed by ESI-MS, and the pure fractions were freeze-dried. Analytical reverse phase C-18-HPLC experiments were performed using a Vydac C_18_ column (250 by 4.6 mm [i.d.]; particle size, 5 μm; 300 Å) and a linear gradient from buffer A to buffer B over 30 min at a flow rate of 1 ml/min.

### Quantification.

The dried powder was dissolved, and the weight was calculated by measuring the UV absorbance using a NanoDrop UV spectrophotometer (extinction coefficient of cyO2, 7,420/M/cm). The purity of cyO2 was more than 95%, as determined by analytical HPLC.

### Antimicrobial peptides.

A mixture of wheat germ histones (WGH) in 0.1% acetic acid was kindly supplied by Lars-Olof Hedén at Lund University, Lund, Sweden (isolated as described previously [51]). CNY100HL (CKYILLLRRQLARAWRRGLR) (52) and LL-37 (LLGDFFRKSKEKIGKEFKRIVQRIKDFLRNLVPRTES) (53) were obtained from Innovagen AB (Lund, Sweden).

### Bacterial strains.

For the serial passage experiment, Salmonella enterica serovar Typhimurium LT2 DA6192 was used. The other strains used are listed in Table SI1 in the supplemental material).

### Serial passaging.

Six independent cultures of *S*. Typhimurium were started in 200 μl of 20 mM sodium phosphate buffer (SPB) with 0.1% Trypticase soy broth (TSB) by adding 10^5^ bacteria to each culture. Four out of the six cultures were treated with cyO2, and the remaining two lineages were untreated controls. For every cycle, 2 μl of an overnight culture was added to a final volume of 200 μl of fresh culture. Every day, samples (60 μl) from each culture were added to a 96-well plate, and the values of the optical density at 620 nm were determined in a plate reader (Multiskan FC; Thermo Scientific). Every 5th day, 120 μl from an overnight culture in 10% dimethyl sulfoxide (DMSO) was frozen at −80°C. CyO2 was added just before addition of bacteria. The starting concentration of cyO2 for the cycling experiment was 6 μM. The concentration of cyO2 was raised by a factor of 1.5 after every 10 cycles. In case the bacteria could not survive in the presence of the higher concentration, cycling from the frozen culture was resumed at a lower concentration for a few more cycles. Cycling was stopped after 150 cycles (900 to 1,050 generations). Ten additional lineages of Salmonella were cycled for 100 cycles.

### Isolation of mutants.

By streaking for single cells, mutant clones were isolated on LA plates. Cultures from these clones were saved in 10% DMSO frozen at −80°C.

### Whole-genome sequencing.

Genomic DNA was isolated using a Genomic Tip 100G DNA kit (Qiagen) according to the manufacturer's instructions. Five resistant clones from the cycling experiment were sent to the Beijing Genome Institute (BGI; China) for whole-genome sequencing using the Illumina sequencing technology. CLC Genomics Workbench software (version 5.5.1; CLC bio, Aarhus, Denmark) was used to assemble the sequencing reads and detect regions with sequence variations (quality-based variant detection) and regions with no coverage compared to the sequence of the *S*. Typhimurium LT2 wild-type strain. The cutoff frequency was set to 75%.

### Time-kill experiments.

Overnight cultures of bacteria in SPB (20 mM) supplemented with 0.1% TSB (Becton, Dickinson and Company, USA) were diluted to 2.5 × 10^6^ to 3.5 × 10^6^ CFU/ml in fresh medium. Ninety microliters of this suspension was transferred into microcentrifuge tubes containing 10 μl of cyO2 solution. The tubes were incubated with shaking at 37°C. Samples (10 μl) were taken from each tube and spread on LA plates at 0, 4 and 24 h at suitable dilutions.

### Determination of antimicrobial peptide MICs.

Overnight cultures of bacteria in SPB (20 mM) supplemented with 0.1% TSB were diluted to 5 × 10^5^ CFU/ml in the same medium. Ninety microliters was transferred into 96-well microtiter plates containing 10 μl of serially diluted cyO2 solution. The plates were incubated in a shaking incubator at 37°C for 16 to 20 h. The tested concentration of cyO2 was 50 μM to 0.39 μM, and 1.5-fold dilution steps were used. At least two independent experiments, each with two technical replicates, were performed.

### Determination of antibiotic MICs.

MIC values for antibiotics were determined using Etests, as described by the manufacturer (AB Biodisk).

### Genetic reconstitution of mutations.

Mutations that appeared in more than one resistant isolate were reconstituted in a wild-type genetic background by standard genetic techniques. In brief, a kanamycin (KanR) or chloramphenicol (ChlR) resistance marker was introduced 3 to 10 kb away from the mutation of interest either by use of a temperature-controlled linear transformation system or by P22 transduction of relevant KanR or ChlR markers from our strain collection. P22 lysates were then prepared with the constructed strains and used to infect the wild-type strain. From each transduction, a congenic strain pair in which one strain carried the mutation and the resistance marker and the other strain carried only the resistance marker was saved. Subsequently, the KanR-ChlR cassette was removed by expression of Flp recombinase, leaving an 85-nucleotide scar. Reconstituted strains were confirmed by PCR and DNA sequencing.

### Fitness measurements in laboratory media.

Fitness measurements were carried out in Mueller-Hinton broth (MHB) and M9 minimal medium plus 0.2% glucose (M9-G). Four independent overnight cultures of bacteria were diluted 1,000-fold in the same media, and 300 μl from this dilution was transferred to 96-well Bioscreen plates (in duplicate). Bacterial exponential growth was measured in a Bioscreen C analyzer (Oy Growth Curves Ab Ltd.) every 4 min for 16 h at 37°C in continuous shaking mode. Relative fitness was determined by dividing the growth rate of mutant strain by that of the congenic wild-type strain.

### Fitness measurements in mice.

Fresh overnight cultures of an *S*. Typhimurium mutant (tagged with the ChlR) and the isogenic wild-type strain (tagged with the KanR) were grown in LB at 37°C and 190 rpm until saturation. The cells were washed 3 times in sterile phosphate-buffered saline (PBS), and the competitors were mixed in a 1:1 ratio. For each competition, four 7-week-old female BALB/c mice were injected intraperitoneally with 100 μl of approximately 2 × 10^6^ to 4 × 10^6^ CFU/ml of bacteria. The number of bacteria in the inoculum was quantified by plating appropriate dilutions on LB agar plates supplemented with chloramphenicol (25 mg/liter) or kanamycin (50 mg/liter). At 2 days postinfection, the mice were sacrificed by cervical dislocation. Spleens and livers were harvested, and homogenized cell suspensions were prepared in PBS. Dilutions were spread on LB plates supplemented with chloramphenicol or kanamycin to determine the ratio of the mutant to the wild type. The competitive index was calculated as the ratio of the mutant to the wild type after competition divided by the ratio of the mutant to the wild type in the initial inoculum. All values were normalized to those of the competition experiment with Salmonella strains tagged with only the ChlR and KanR. The Uppsala Animal Ethics Committee (permit no. C154/14) approved all animal experiments.

### Statistical analysis.

The statistical significance of the fitness measurements in laboratory growth medium was determined by Student's *t* test, with the significance level being set to 95% (*P* < 0.05). In the *in vivo* fitness assay, the median values were used, while the data for all the replicates are shown. Data for time-kill assays and MIC assays represent the average values from at least two independent experiments.

## Supplementary Material

Supplemental material
