# Supplementary material for: Resistance to the Cyclotide Cycloviolacin O2 in Salmonella enterica Caused by Different Mutations That Often Confer Cross-Resistance or Collateral Sensitivity to Other Antimicrobial Peptides
Source: Antimicrob Agents Chemother. 2017 Jul 25;61(8):e00684-17. doi: 10.1128/AAC.00684-17 (PMC5527591; doi:10.1128/AAC.00684-17)
Supplement: Supplemental material [file supp_61_8_e00684-17__index.html]

Supplemental material 

# Resistance to the Cyclotide Cycloviolacin O2 in Salmonella enterica Caused by Different Mutations That Often Confer Cross-Resistance or Collateral Sensitivity to Other Antimicrobial Peptides

## Supplemental material

- Supplemental file 1 -

  Tables S1 to S5 and Fig. S1

  PDF, 336K
