# Supplementary material for: Resistance to the Cyclotide Cycloviolacin O2 in Salmonella enterica Caused by Different Mutations That Often Confer Cross-Resistance or Collateral Sensitivity to Other Antimicrobial Peptides
Source: Antimicrob Agents Chemother. 2017 Jul 25;61(8):e00684-17. doi: 10.1128/AAC.00684-17 (PMC5527591; doi:10.1128/AAC.00684-17)

Supplementary Information

Tables

Table SI-1. List, genotype and origin of strains used in this study.

Strain	Genotype, description	Origin
DA6192	Wild type LT2	Lab collection
DA34444	Sty LT2 cycloviolacin O2 resistant (isolated)	This work
DA34445	Sty LT2 cycloviolacin O2 resistant (isolated)	This work
DA34446	Sty LT2 cycloviolacin O2 resistant (isolated)	This work
DA34447	Sty LT2 cycloviolacin O2 resistant (isolated)	This work
DA34448	Sty LT2 cycloviolacin O2 resistant (isolated)	This work
DA34449	Eco K12 MG1655 cycloviolacin O2 resistant (isolated)	This work
DA34450	Eco K12 MG1655 cycloviolacin O2 resistant (isolated)	This work
DA34451	Sty LT2, *pmrA*:552A>C, FRT scar	This work
DA34452	Sty LT2, *pmrA* wild type, FRT scar	This work
DA34453	Sty LT2, *phoQ*:1082T>G, FRT scar	This work
DA34454	Sty LT2, *phoQ* wild type, FRT scar	This work
DA34455	Sty LT2, *yjeP*:1684A>G, FRT scar	This work
DA34456	Sty LT2, *yjeP* wild type, FRT scar	This work
DA34457	Sty LT2, *rnc*:512T>A, FRT scar	This work
DA34458	Sty LT2, *rnc* wild type, FRT scar	This work
DA34459	Sty LT2, *ftsW*:743C>A, FRT scar	This work
DA34460	Sty LT2, *ftsW* wild type, FRT scar	This work
DA34461	Sty LT2, *plsX*:272T>A, FRT scar	This work
DA34462	Sty LT2, *plsX* wild type, FRT scar	This work
DA38416	Sty LT2, *envZ*:251T>A, FRT scar	This work
DA38417	Sty LT2, *envZ* wild type, FRT scar	This work
DA38418	Sty LT2, *pmrA*:567delT, FRT scar	This work
DA38419	Sty LT2, *pmrA* wild type, FRT scar	This work

DA39973	Sty LT2, *rpoC*:3058T>A, FRT scar	This work
DA39975	Sty LT2, *rpoC* wild type, FRT scar	This work
DA39977	Sty LT2, *pnp*:1631C>A, FRT scar	This work
DA39979	Sty LT2, *pnp* wild type, FRT scar	This work
DA38457	Sty LT2, *ftsW*:743C>A, FRT scar, *yjeP*:1684A>G, FRT scar	This work
DA38459	Sty LT2, *ftsW*:743C>A, FRT scar, *rnc*:512T>A, FRT scar	This work
DA38461	Sty LT2, *ftsW*:743C>A, FRT scar, *plsX*:272T>A, FRT scar	This work
DA38894	Sty LT2, *ftsW*:743C>A, FRT scar, *pmrA*:567delT, FRT scar	This work
DA39029	Sty LT2, *ftsW*:743C>A, FRT scar, *phoQ*:1082T>G, FRT scar	This work
DA39033	Sty LT2, *ftsW*:743C>A, FRT scar, *envZ*:251T>A, FRT scar	This work
DA39980	Sty LT2, *ftsW*:743C>A, FRT scar, *rpoC*:3058T>A, FRT scar	This work
DA39986	Sty LT2, *ftsW*:743C>A, FRT scar, *pnp*:1631C>A, FRT scar	This work
DA46882	Sty LT2, *phoQ*:1082T>G, FRT scar; *pmrA*:552A>C, FRT scar	This work
DA46883	Sty LT2, *phoQ*:1082T>G, FRT scar; *pmrA*:567delT, FRT scar	This work
DA23175	Sty LT2, pmrB(R13H)	Lofton *et. al.*, 2013
DA23307	Sty LT2, phoP(D23N)	Lofton *et. al.*, 2013
DA22427	Sty LT2, waaY(del bp17RFS)	Lofton *et. al.*, 2013
DA23899	Sty LT2, waaY(del bp17RFS), pmrB(R13H), phoP(D23N)	Lofton *et. al.*, 2013
DA12088	Sty LT2, Δ*sbmA*, PR-39 resistant	Pränting *et. al.*, 2008
DA11799	Sty LT2, *hemL* tgg→cgg, protamine resistant	Pränting & Andersson, 2010
DA10848	Sty LT2, *pmrB* ACG→CCG, colistin resistant	Song & Andersson, 2009
DA10857	Sty LT2, *pmrA* CGC→TGC, colistin resistant	Song & Andersson, 2009
DA16875	Wheat germ histone resistant mutant	Lofton *et. al.*, 2013
DA16874	Sty LT2, LL-37 resistant mutant	Lofton *et. al.*, 2013
DA17610	Sty LT2, CNY100HL resistant mutant	Lofton *et. al.*, 2013

Table SI-2. List of mutations in the isolated cyO2 resistant mutants.

Strain	Genes with single nucleotide polymorphisms	Genes with deletion/insertions
DA34444	*ftsW, phoQ, STM2689 (pseudogene), envZ, pmrA, yjeP*	*malT* (promoter)
DA34445	*ftsW, plsX, rnc, pnp, rpoC, serS* (promoter)	*yeaG, malT* (promoter), *pmrA*
DA34446	*ybjG, mdoH, perM, uppP, malT, rpoC, rrsE*	*rrsH, stpA, sufI*
DA34447	*ftsW, STM0285, plsX, dedD, rnc, pnp, malT, yhjU, wecC, rpoC, serS* (promoter)	*crI, pmrA*
DA34448	*ftsW, ftsW, rpsA, RBS, yabI, nlpI, malT, pitA, rcsC, pmrB* (terminator)	*mdoB, hflK, ptsP*

Table SI-3. Genes with mutations in more than one isolated cycloviolacin O2 resistant mutants.*

**Gene**	**Mutation**	**Gene function**
ftsW	743C>A (T248N)**	Cell division protein
envZ	251T>A (L84Q)	Membrane localized osmosensor component of envZ/ompR system
pmrA	567delT (T189fs)***	Transcriptional regulatory component of pmrAB system
yjeP	1684A>G (T562A)	Mechanosensitive ion channel
rnc	512T>A (L171Q)	Ribonuclease III, processes rRNAs and some mRNAs
rpoC	3058T>A (W1020R)***	DNA dependent RNA polymerase
pnp	1631C>A (P544Q)	Polynucleotide phosphorylase, member of mRNA degradosome
plsX	272T>A (L91Q)	Role in fatty acid and phospholipid biosynthesis
malT	1146G>A (W382*)***	Positive regulation of maltose uptake and catabolism

*All of these mutations (except *malT*), were reconstituted in a wild type background. Another mutation *phoQ*:1082T>G (V361G), was also reconstituted though it appeared in only one isolated mutant.
**One of the sequenced strain contained two mutations in *ftsW*.
***One of the sequenced strains contained a different substitution instead of the mentioned mutation.

Table SI-4. MIC values of cyO2 resistant mutants against different antimicrobial peptides.*

Strain	cyO2 [µM]	LL-37 [mg/L]	CNY [mg/L]	WGH [mg/L]
wild type	8.1±1.7	8.7±1.8	1.9±0.9	5±0
ftsW (T248N)	10.2±1.1	10±0	2.5±0	10±0
envZ (L84Q)	9.9±0.9	10±0	2.5±0	8.7±1.8
phoQ (V361G)	13.5±4.8	5±0	3.1±0.8	10±0
pmrA (T189fs)	12±0.9	7.5±3.5	3.7±1.8	10±0
pmrA (E148D)	9.4±0	7.5±3.5	2.5±0	10±0
yjeP (T562A)	11.5±1.8	10±0	2.5±0	10±0
rnc (L171Q)	9.4±4.1	10±0	2.5±0	8.7±1.8
rpoC (W1020R)	9.4±0	ND	2.5±0	10±0
pnp (P544Q)	13.4±5.7	ND	5±0	10±0
plsX (L91Q)	4.3±2.7	ND	2.5±0	10±0

*For cyO2, three independent experiments were done in duplicates. For the other peptides, two independent experiments were done in duplicates.

Table SI-5. MIC values of antibiotics (determined by E test) against cyO2 resistant strains.

Antibiotics	wt	*ftsW*	*envZ*	*phoQ*	*pmrA* (fs)	*pmrA* (sub)	*yjeP*	*rnc*	*rpoC*	*pnp*	*plsX*
Colistin	0.125	0.125	0125	0.094/ 0.125	0.125	0.094/ 0.125	0.094/ 0.125	0.125	0.125	0.125	0.125
Polymyxin B	0.19	0.19	0.19	0.19	0.19	0.19	0.19	0.19	0.19	0.19	0.19
Penicillin G	16	8	>32	>32	8	12	12	32	12	8	12
Ampicillin	1	0.75	2	0.75	ND	0.75	0.75	0.75	ND	0.75	1
Piperacillin	1.5	1.5	ND	ND	ND	ND	ND	ND	ND	ND	ND
Cefotaxime	0.064	0.064	ND	0.094	ND	0.094	0.064	0.064	0.064	0.094	0.125
Imipenem	0.19	0.25	ND	0.25	ND	0.25	0.19	0.25	ND	ND	0.25
Ertapenem	0.004	0.006	0.006	ND	0.006	ND	ND	ND	0.004	0.003	ND
Ciprofloxacin	0.012	0.012	0.023	0.012	ND	0.012	0.012	0.012	0.012	0.008	0.016
Tetracycline	0.75	1	1	1	1	1	1	0.75	0.75	0.75	1
Tigecycline	0.094	0.094	0.125	0.094	0.064	0.064	0.064	0.094	0.094	0.094	0.094
Trimet-Sulfa	0.023	0.047	ND	0.032	ND	0.023	0.047	0.032	ND	ND	0.032
Chloramphenicol	4	2	4	3	ND	3	2	3	2	2	2
Rifampicin	8	12	12	8	8	8	8	6	8	6	12
Erythromycin	12	16	32	16	ND	8	16	16	24	24	16
Kanamycin	1	1.5	1.5	1.5	ND	1.5	1.5	1.5	1.5	1	1.5
Gentamicin	0.19	0.19	ND	0.19	ND	0.25	0.25	0.19	ND	ND	0.19
Steptomycin	2	3	4	4	ND	4	4	3	4	4	6
Phosphomycin	0.19	ND	ND	0.19	ND	0.19	0.19	ND	0.064	0.094	ND
Linezolid	ND	>256	ND	>256	ND	>256	>256	>256	ND	ND	>256
Fusidic acid	ND	>256	>256	>256	>256	>256	>256	>256	ND	ND	>256

Figure SI-1. Cross resistance of cyO2 resistant strains to cyO3.

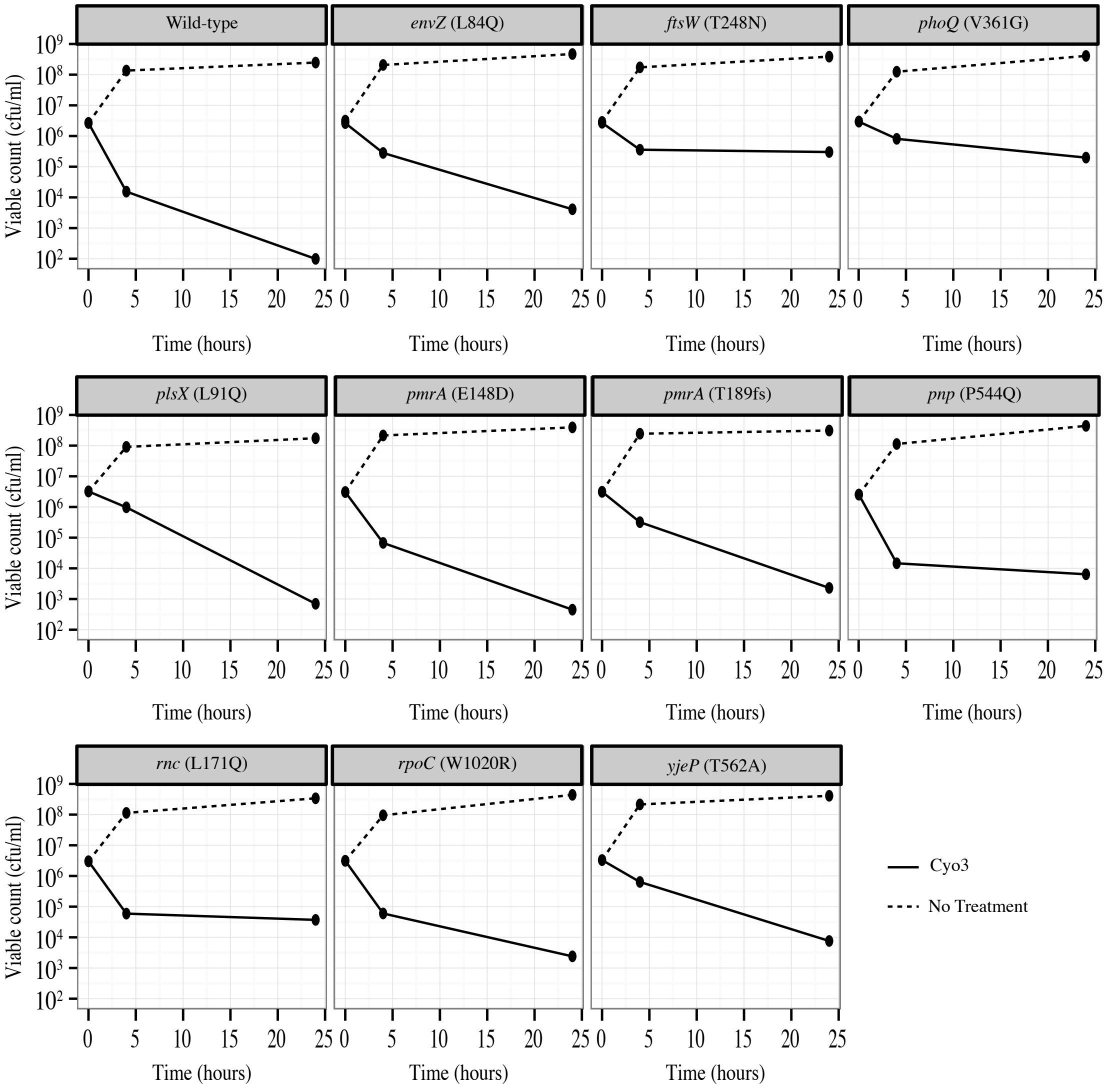

Supplement: Supplemental material [file AAC.00684-17_zac009176441s1.pdf]
